# A quantitative CT parameter for the assessment of pulmonary oedema in patients with acute respiratory distress syndrome

**DOI:** 10.1371/journal.pone.0241590

**Published:** 2020-11-12

**Authors:** Patrick Leiser, Thomas Kirschning, Christel Weiß, Michael Hagmann, Jochen Schoettler, Franz-Simon Centner, Holger Haubenreisser, Philipp Riffel, Sonja Janssen, Claudia Henzler, Thomas Henzler, Stefan Schoenberg, Daniel Overhoff

**Affiliations:** 1 Institute of Clinical Radiology and Nuclear Medicine, University Medical Centre Mannheim, Heidelberg University, Mannheim, Germany; 2 Department of Anaesthesiology and Intensive Care Medicine, University Medical Centre Mannheim, Heidelberg University, Mannheim, Germany; 3 Department for Medical Statistics and Biomathematics, University Medical Centre Mannheim, Heidelberg University, Mannheim, Germany; San Gerardo Hospital, ITALY

## Abstract

**Objectives:**

The aim of this study was to establish quantitative CT (qCT) parameters for pathophysiological understanding and clinical use in patients with acute respiratory distress syndrome (ARDS). The most promising parameter is introduced.

**Materials and methods:**

28 intubated patients with ARDS obtained a conventional CT scan in end-expiratory breathhold within the first 48 hours after admission to intensive care unit (ICU). Following manual segmentation, 137 volume- and lung weight-associated qCT parameters were correlated with 71 clinical parameters such as blood gases, applied ventilation pressures, pulse contour cardiac output measurements and established status and prognosis scores (SOFA, SAPS II).

**Results:**

Of all examined qCT parameters, excess lung weight (ELW), i.e. the difference between a patient’s current lung weight and the virtual lung weight of a healthy person at the same height, displayed the most significant results.

ELW correlated significantly with the amount of inflated lung tissue [%] (p<0.0001; r = -0.66) and was closely associated with the amount of extravascular lung water (EVLW) (p<0.0001; r = 0.72). More substantially than the oxygenation index (PaO_2_/FiO_2_) or any other clinical parameter it correlated with the patients’ mean SOFA- (p<0.0001, r = 0.69) and SAPS II-Score (p = 0.0005, r = 0.62). Patients who did not survive intensive care treatment displayed higher values of ELW in the initial CT scans.

**Conclusions:**

ELW could serve as a non-invasive method to quantify the amount of pulmonary oedema. It might serve as an early radiological marker of severity in patients with ARDS.

## Introduction

ARDS constitutes a very heterogeneous syndrome. It can be caused by various pulmonary and extrapulmonary triggering factors, the most common being pneumonia and sepsis. This heterogeneity of underlying causes has hampered the search for a comprehensive and uniform classification as well as the implementation of quantitative CT (qCT) analysis.

Under the assumption that lung density differs within the range of gas (-1000 Hounsfield Units, HU) and water (0 HU), each voxel can be assigned to a certain degree of aeration [1]. The selection of multiple voxels then allows the measurement of various volume- or lung weight-associated parameters [1]. Furthermore, thresholds were defined for the differentiation between not aerated (>-100 HU), poorly aerated (-100 HU to -500 HU), well aerated (-500 HU to -900 HU) and over-aerated lung tissue (<-900 HU) [1] and used to describe the distribution of atelectasis and emphysema [2]. Dynamic CT scans then enabled the quantitative analysis of recruitment and derecruitment throughout the ventilation cycle [3]. This led to an increased knowledge on ARDS pathophysiology revealing disease modifying factors such as gravity [4], positive end-expiratory pressure (PEEP) [5] and prone positioning [6].

In the clinical setting, physiology scores like ‘Sequential Organ Failure Assessment Score’ (SOFA) or ‘Simplified Acute Physiology Score II’ (SAPS II) gained increasing importance within the last decades [7, 8]. Assigning the degree of multiple organ dysfunction to a certain score, they provide a good overview on a patient’s pathophysiological state and are linked to mortality. Especially the mean score of SOFA (SOFA mean), i.e. SOFA averaged over the entire stay on intensive care unit (ICU), revealed the closest association to patient mortality [9, 10].

However, throughout most of the previous studies, approaching ARDS by quantitative CT analysis, the translatability of radiological parameters into clinical routine and their relation to clinical measurements remained vague. Therefore, the aim of this study was not only to develop quantitative CT parameters but also to validate them by a correlation with associated clinical parameters and, where possible, to elaborate their physiological correlate. The parameters were thought to serve as surrogate parameters for both research purposes and as a fast and non-invasive method to determine a patient’s status in clinical practice.

## Materials and methods

This study was approved by the local ethics committee 'Medizinische Ethikkommission II, University Medical Centre Mannheim, Heidelberg University, Germany'. Written informed consent was obtained from each patient or their authorised representative.

Following approval by the local ethics committee and written informed consent of each patient or their authorised representative, 36 patients with suspected ARDS who were admitted to our intensive care unit between November 2016 and January 2018 were included in this prospective study. The study was performed in adherence to the Declaration of Helsinki.

Exclusion criteria for the study were: age younger than 18 years, pregnancy, severe head injury, inherited cardiac malformations, presence of arrhythmias, immunosuppression, end-stage chronic organ failure, expected survival of less than 24 hours and failure to meet the ARDS criteria according to the Berlin definition [11] in the sequel.

As a consequence, the initial amount of 36 patients with suspected ARDS decreased to a study population of 28 patients (20 male, 8 female) with a mean age of 58 ± 13.4 years.

### Image acquisition

Accordingly, 28 intubated patients with diagnosed ARDS compliant with the Berlin definition received a non-contrast enhanced chest CT within the first 48 hours on our intensive care unit. In order to create comparable data, all CT scans were performed during a breathhold in end-expiration with an underlying positive end-expiratory pressure according to individual PEEP trial (see S1 File for more details).

Images were acquired using a second generation dual source CT scanner (Somatom Definition Flash) with 32 × 0.6 mm collimation, 89/76 reference mAs at 120 kV, pitch of 0.8 and 0.5 sec rotation time. Furthermore, a slice thickness of 1.5 mm, increment of 1.2 mm and medium-soft convolution kernels (I31f; Q33f) were chosen for all CT acquisitions.

### Image analysis/ Creation of quantitative CT parameters

CT scans were analysed semiautomatically using dedicated software (syngo.via, Siemens, Germany). However, since atelectatic lung was often misclassified as extrapulmonary soft tissue, manual lung segmentation was regularly necessary. In patients with mild to moderate ARDS according to the Berlin definition [11] this took 16 mins on average, yet in patients with severe ARDS this process lasted up to 2.5 hours. Subsequently, quantitative CT parameters were calculated for each patient and allocated to one of the two groups: inflation or lung weight parameters. The list of all collected parameters and their derivation can be found in (S2 Table in S2 File). In the following, we have confined ourselves to the brief explanation of only those parameters that were of major significance for the ongoing study.

#### Inflation

We defined the amount of inflated lung tissue [%] as the sum of well inflated and poorly inflated tissue divided by total lung volume.

$$inflated\;lung\;tissue\;[\%]=\frac{well\;inflated\;tissue\;[ml]+poorly\;inflated\;tissue\;[ml]}{total\;lung\;volume\;[ml]}$$

#### Lung weight

To our knowledge Mull et al. [12] were the first to describe the relation between CT number [HU] and physical density (ρ) [g/cm^3^] which for frozen meat (0 HU; 1,02g/cm^3^) almost resembled water (0 HU; 1g/cm^3^). With that knowledge and assuming that human lung only consist of water/soft tissue (0 HU) and gas (-1000 HU), it is possible to compute lung weight from a given gas and tissue volume [13]. Cressoni et al. developed a formula for the estimation of lung weight based on height in elderly people (age 64 ±13 years) [14]. We used this formula to calculate the excess of lung weight (ELW) in our patients.

estimatedlungweight[g]=−1806.1+1633.7×subject’sheight[m]

excesslungweight=lungmass[g]−estimatedlungweight[g]

#### Clinical parameters

Next to these radiological parameters we collected clinical data including blood gases, ventilator settings and pulse contour cardiac output (PiCCO®) measurements (for a full list see S2 Table in S2 File) at the time of CT acquisition. Among the PiCCO measurements the parameter ‘extravascular lung water’ (EVLW) needs to be emphasised. EVLW is determined by transpulmonary thermodilution and serves as a method to estimate the amount of extravascular lung water, i.e. pulmonary oedema on intensive care units [15]. Moreover, status and prognosis scores (SOFA, SAPS II) were determined for every patient of our study and on each day throughout their stay on ICU [7, 8].

### Statistical analysis

All generated qCT parameters underwent a comprehensive statistical analysis using dedicated software (Statistical Analysis System, SAS 9.4, Cary, North Carolina).

In a first step, quantitative CT parameters of each group (inflation and lung weight) were correlated among themselves and with related clinical parameters. The aim was to determine the qCT parameter which best represents each group. In a second step, the most representative qCT parameter of each group was correlated with radiological and clinical parameters of the other group in order to reveal pathophysiological connections.

In cases of a strong correlation, intra-class correlation coefficient (ICC)- and Bland-Altman analysis were conducted to examine the level of concordance between the parameters [16].

## Results

The majority of the 28 patients of our study developed ARDS due to infectious complications (13 pneumonia, 9 sepsis, 5 aspiration, 1 near-drowning). In accordance with the Berlin definition [11] we diagnosed two mild, 19 moderate and 7 severe cases of ARDS. On average, each patient spent 22 days under mechanical ventilation on our ICU receiving volume-controlled ventilation with a tidal volume of approx. 6 ml/kg ideal body weight (IBW) and an average PEEP of 13.2 ± 4.1cm H_2_O. Furthermore, patients were treated with a slightly more liberal fluid balance as the averaged PiCCO measurements collected at the time of CT acquisition (cardiac index (CI) = 3.6 ± 1 l/min/m^2^; intrathoracic blood volume index (ITBVI) = 900 ± 183 ml/m^2^ and extravascular lung water index (EVLWI) = 14.6 ± 6.2 ml/m^2^) reveal. Seven patients required extracorporeal membrane oxygenation (ECMO) therapy. A survival of 15 patients corresponds to a mortality of 46%.

A characterisation of our study population is shown in S2 Table in S2 File. On average the group of male patients (n = 20) displayed significantly higher total lung volumes than the female subpopulation (n = 8). The slightly decreased PaO_2_/FiO_2_ and the increased SOFA _mean_ and SAPS II _mean_-scores in the group of male patients were accompanied by a higher amount of over-inflated tissue [%] and a higher excess in lung weight [g].

For reasons of clarity we have concentrated on those parameters that were of major significance for the study with a strong emphasis on lung weight parameters and the qCT parameter ‘excess lung weight’ (ELW).

### Lung weight analysis

Of all analysed lung weight parameters excess lung weight (i.e. the surplus of lung weight in comparison to the estimated lung weight according to Cressoni et al. [14] displayed the best results in correlation with parameters of other groups (Table 1).

**Table 1:** Comparison of excess lung weight and PaO_2_/FiO_2_ in correlation with parameters of other groups.

Parameter	Excess lung weight		PaO_2_/FiO_2_	
	r	P value	r	P value
**Inflation/oxygenation:**				
Inflated tissue [%]	**-0.66**	**0.0001**	0.11	0.57
Well inflated tissue [%]	**-0.59**	**0.001**	0.07	0.74
SOFA_lung _mean_	**0.35**	**0.06**	-0.12	0.53
SOFA_lung _CT_	0.13	0.5	**-0.27**	**0.18**
PaO_2 CT_ [mmHg]*	0.08	0.7	**0.58**	**0.001**
PaCO_2_ [mmHg]*	0.06	0.78	**-0.41**	**0.03**
**Haemodynamics:**				
SOFA_cv _mean_	**0.41**	**0.03**	0.07	0.73
SOFA_cv _CT_	**0.3**	**0.12**	-0.54	0.79
**PiCCO**^**®**^ **measurements:**				
EVLW [ml]	**0.72**	**<0.0001**	-0.16	0.45
EVLWI [ml/m^2^]	**0.68**	**0.0001**	-0.13	0.54
**Applied ventilation pressures:**				
PEEP [mmHg]*	**0.37**	**0.05**	-0.08	0.67
Pdrive [mmHg]*	-0.31	0.11	**-0.35**	**0.07**
Ppeak [mmHg]*	-0.04	0.83	**-0.46**	**0.01**
**Status and prognosis scores:**				
SOFA _mean_	**0.69**	**<0.0001**	0.08	0.68
SAPS II _mean_	**0.62**	**0.0005**	-0.22	0.27
SOFA _CT_	**0.55**	**0.003**	-0.04	0.85
SAPS II _CT_	**0.59**	**0.001**	-0.25	0.21
SOFA _initial_	**0.57**	**0.0015**	0.09	0.66

Abbreviations: r = correlation coefficient; PaO_2_ = arterial partial pressure of oxygen; FiO_2_ = fraction of inspiratory oxygen; SOFA = Sequential Organ Failure Assessment Score; PaCO_2_ = arterial partial pressure of carbon dioxide, EVLW = extravascular lung water; EVLWI = extravascular lung water index; PEEP = positive end-expiratory pressure; Pdrive = driving pressure; Ppeak = peak (inspiratory) pressure; SAPS II = Simplified Acute Physiology Score II. Parameters were either collected on admission to intensive care unit (initial) or at the time of CT acquisition (CT). ‘Mean’ indicates an average score over a patient’s stay on ICU. ‘Lung’ and ‘cv’ represent the respiratory and cardiovascular subcategory of SOFA. The respective stronger correlation is displayed in bold.

*Calculations and correlations were conducted in mmHg (1mmHg ≈ 133.32 Pa).

Excess lung weight (ELW) showed highly significant correlation with parameters describing inflation (inflated lung tissue [%] p = 0.0001 r = -0.65), haemodynamics (SOFA_cv _mean_ p = 0.03 r = 0.41) and applied ventilation pressures (PEEP [mmHg] p = 0.05 r = 0.37).

**Fig 1:**
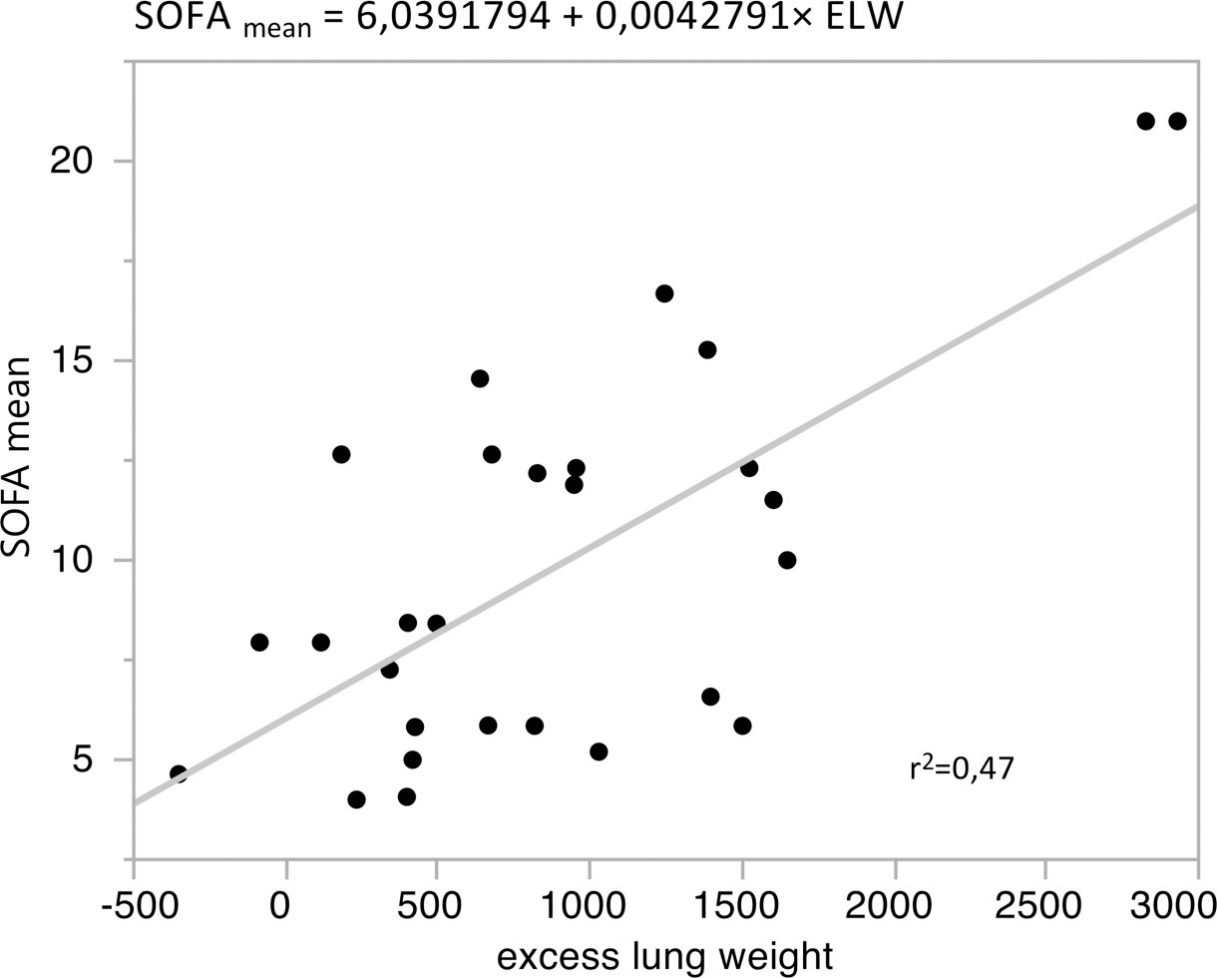
Correlation between excess lung weight and SOFA _mean_. p <0.0001; r = 0.69.

Correlated to status and prognosis scores, ELW displayed very strong connections to SOFA _mean_ (p<0.0001 r = 0.69) and SAPS II _mean_ (p = 0.0005 r = 0.62). The correlation with the mean score of SOFA and SAPS II was even higher than correlation with the score from the day of CT acquisition (Fig 1; Table 1).

#### Intra-class correlation coefficient (ICC) and Bland-Altman analysis

The strongest association with excess lung weight was observed in the correlation with extravascular lung water (EVLW) (r = 0.71, p<0.0001) a PiCCO measurement determined by transpulmonary thermodilution (Table 1).

**Fig 2:**
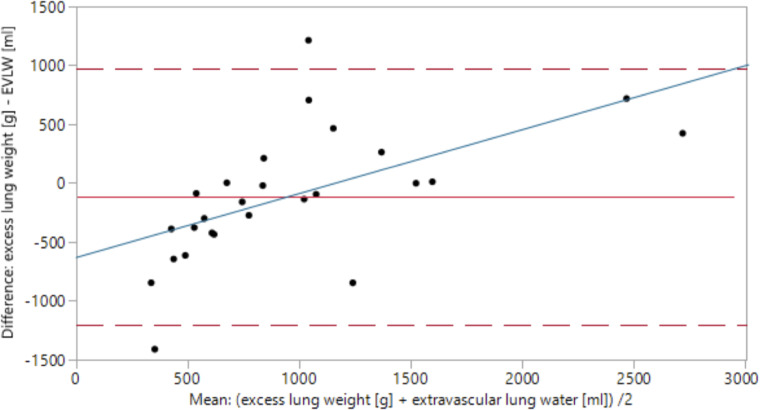
Bland-Altman analysis: Excess lung weight–extravascular lung water. The x-axis represents the average of associated values of excess lung weight (ELW) and extravascular lung water (EVLW); (ELW + EVLW)/2. The y-axis represents the difference (ELW—EVLW). The mean difference between ELW and EVLW equals -116.6 g (red line). The dashed red lines mark the 95%-confidence interval. y = 0 represents the borderline between overestimation (y>0) and underestimation (y<0) of EVLW by ELW. Over- and underestimation are dependent on the range of measurement (p = 0.002), a trend visualised by the blue regression line.

Due to this very strong connection between ELW and extravascular lung water (EVLW) (r = 0.71, p<0.0001), we performed a combined ICC- and Bland-Altman analysis to determine their level of concordance (Fig 2).

On average, ELW underestimated EVLW by 116.6 ± 112.4 g with a mean EVLW of 1021.2 ml within the study population. A paired t-test did not detect a significant difference between these mean values (p = 0.3). However, an ICC of 64.7% suggests that 36.3% of all values’ discrepancies are attributable to differences between ELW and EVLW. Over- and underestimation are dependent on the range of measurement (p = 0.002). While ELW predominantly underestimates EVLW at values below approx. 1150 g, values above 1150 g are associated with a tendency of overestimation.

#### Comparison between excess lung weight and PaO_2_/FiO_2_

The results of the comparison between excess lung weight and PaO_2_/FiO_2_ both collected at the time of CT acquisition can be examined in Table 1.

#### Comparison between excess lung weight and patient survival

**Fig 3:**
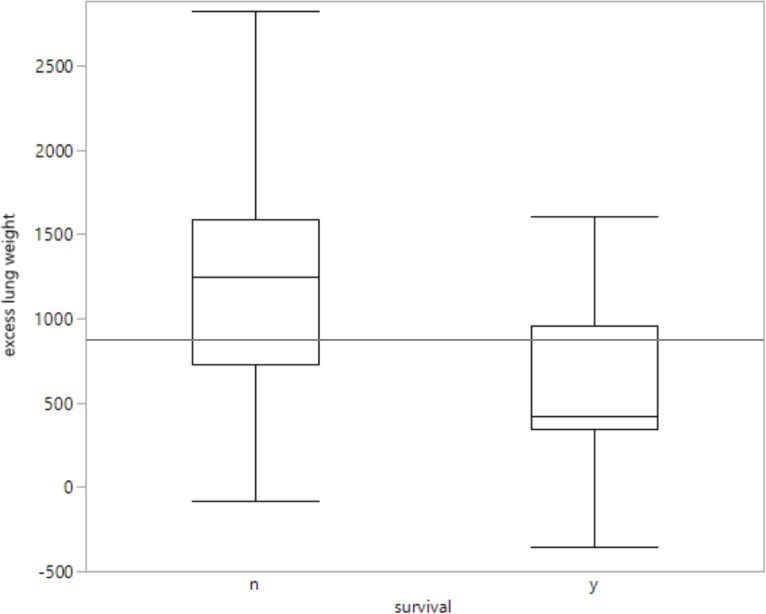
Comparison between excess lung weight and patient survival. n = no survival, i.e. death during hospitalisation (n = 13); y = yes, i.e. survival during ICU and the subsequent hospitalisation phase (n = 15). The grey line (y = 899.3 g) indicates the mean value of excess lung weight over the total study population.

The 15 patients who survived treatment on intensive care unit displayed a median ELW of 425 g, while the thirteen patients who died during hospitalisation presented a median ELW of 1245 g and a 25% quantile of 728 g (Fig 3). Two outliers with a very high value of ELW (2869 g and 2931 g) both belong to the group of non-survivors.

## Discussion

**Fig 4:**
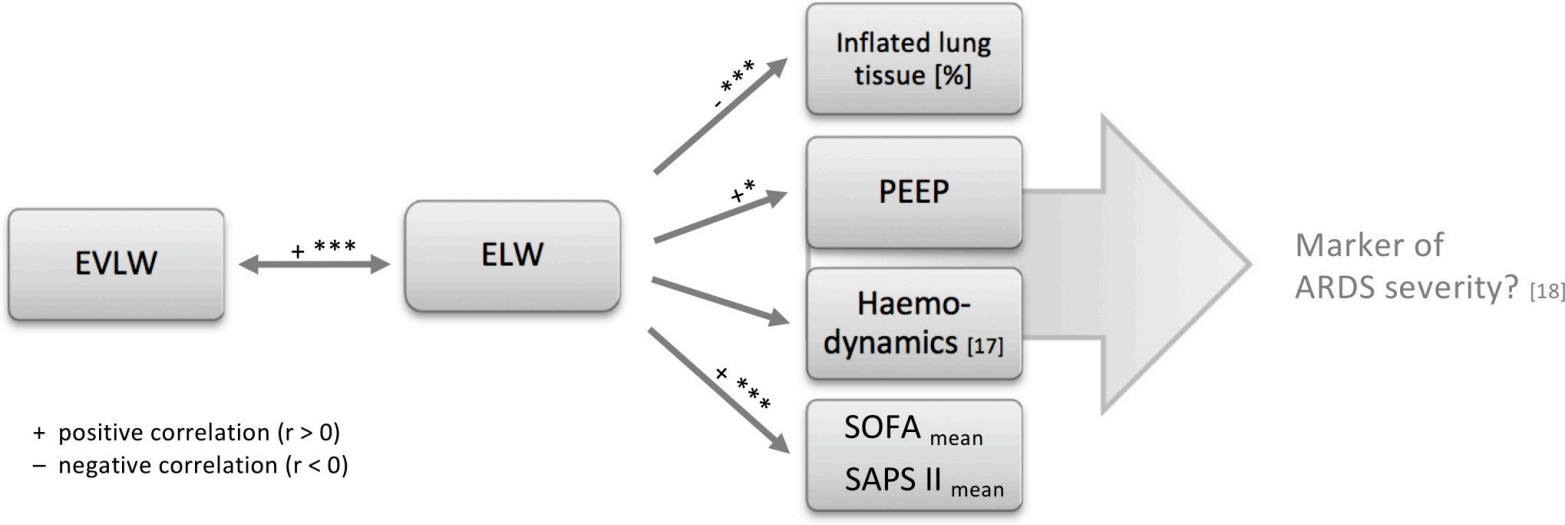
Excess lung weight in the pathophysiology of ARDS. ELW = excess lung weight; EVLW = extravascular lung water; PEEP = positive end-expiratory pressure, SOFA, Sequential Organ Failure Assessment Score; SAPS II, Simplified Acute Physiology Score II. * = p ≤0.05; ** = p ≤0.01; *** = p ≤0.0001.

On the basis of our results we created a central illustration which summarises the main pathophysiological connections elaborated in this study (Fig 4).

### Lung weight

Lung weight seems to have a major influence on various important aspects of pathophysiology in ARDS. It interferes with pulmonary function (inflated lung tissue [%]) and is associated with higher PEEP levels necessary to maintain an adequate functional residual capacity (fRC). The increase in intrathoracic pressure also bears circulatory effects, such as a diminished venous return [17] and an increased demand for catecholamines (SOFA_cv _mean_). Consequently, ELW was also strongly associated with a patient’s overall physiological state (SOFA, SAPS II). This corresponds to recent findings by Maiolo et al. who reported that patients with a more severe ARDS regularly reveal higher amounts of lung weight [18].

Interestingly, in this study the strongest correlation of ELW existed with the average scores of SOFA and SAPS II (SOFA/SAPS II _mean_, Table 1). This circumstance that a parameter collected within the first 48 hours after admission reveals highly significant correlation with status scores averaged over the entire subsequent stay on ICU, implies a certain prognostic value. Especially with regards to the findings by Baradari et al. who stated that a patient’s mean SOFA score is the best indicator for patient mortality on ICU [10]. This complies with our findings presented in Fig 3. Patients who survived the hospitalisation phase displayed lower values of ELW in the initial CT scan. However, due to the small study population these results need to be confirmed on a larger study group before a direct connection between ELW and patient mortality can be postulated (Fig 3).

#### Relation between ELW and EVLW

Due to the strong correlation between excess lung weight and various parts of ARDS pathophysiology the question for its physiological correlate arose. Supported by the strong correlation to EVLW (Table 1), we concluded that ELW represents a CT-based, non-invasive way to quantify the amount of lung oedema.

However, a complete congruence of ELW and EVLW could not be derived from our data (Fig 2). Possible reasons could be inaccuracies in the determination of either of them: Due to its determination by single transpulmonary thermodilution, EVLW shows a comparatively high fluctuation between each measurement. ELW, on the other hand, is highly dependent on precise lung segmentation.

Several studies rated single thermodilution technique as ‘accurate enough’ for the estimation of EVLW in clinical practice [15, 19–21]. However, a dependence on various factors such as age, PaO_2_/FiO_2_, tidal volume and PEEP has been described [21]. In a study by Sakka et al. [20], the determination of EVLW by single thermodilution technique was compared to the gold standard of double indicator-derived EVLW [20]. As a result, thermodilution EVLW systematically overestimated double indicator-derived EVLW at low-normal values and underestimated the gold standard EVLW at higher values (>12ml/kg) [15].

This coincides well with our findings (Fig 2) and would imply that ELW constitutes the more accurate method of quantifying lung oedema. Further studies such as a comparison of ELW and the double indicator-based method should be conducted to confirm these findings and to enable an implementation of ELW into clinical routine.

#### Comparison between excess lung weight and PaO_2_/FiO_2_

Based on the potential of lung weight to interfere with various dimensions of ARDS pathophysiology, the question arose whether ELW would be a suitable supplement for the classification of ARDS. Currently, the oxygenation index PaO_2_/FiO_2_ constitutes the parameter of choice to differentiate between mild, moderate and severe ARDS [11].

As depicted in (Table 1), PaO_2_/FiO_2_ primarily correlates with the patient’s current oxygenation (PaO_2_) and acute lung status (SOFA_lung _CT_), while ELW describes the more persisting impact on the latter (SOFA_lung _mean_). Moreover, ELW seems to be superior to PaO_2_/FiO_2_ in assessing the overall status and prognosis (SOFA _mean_, SAPS II _mean_) as well as main physiological parameters (inflated lung tissue [%], PEEP, SOFA_cv _mean_). This superiority may be subject to ELW’s pathophysiological equivalent: Lung oedema seems to be the disease sustaining factor and as such it can predict a patient’s clinical course much better than any information on the patient’s current oxygenation status could [22].

Consequently, ELW could serve as an early radiological marker during the first hours of ICU treatment and deliver useful extra information to the PaO_2_/FiO_2_-based classification of ARDS severity [11]. However, it must be stated that a widespread implementation of CT scans in these patients is limited by inadequate imaging facilities in smaller hospitals and the risk of transporting these patients to the CT scanner.

### Limitations of the study

A general limitation of this study is the limited number of patients enrolled. This was partly due to the fact that some patients who were initially enrolled in the study did not meet the ARDS definition criteria in the sequel. In case of the introduced parameter ELW, differentiation between significant and insignificant results was mostly unambiguous (Table 1). However, the correlation between ELW and SOFA _mean_ appeared sensitive to two outliers (ELW value close to 3000g; Fig 1). If these are removed the remaining correlation is less strong (intersection = 6.95; slope = 0.0028; r^2^ = 0.17; r = 0.41) yet still significant (p = 0.038).

Consequently, further investigations will be necessary to reduce the effect of potential biasing factors (e.g. outliers) and to re-evaluate the connection to patient mortality in the light of a greater study population. Only once these results are confirmed, an implementation of ELW into clinical practice can take place.

A second limitation of this study concerns the derivation of ‘excess lung weight’. ELW founds on the research by Cressoni et al. who used data from 100 CT scans performed in end-inspiratory breathhold to develop a formula for an estimation of lung weight based on people’s height [14]. The bias resulting from the differing breathhold manoeuvres can be regarded as negligible considering that at 37°C two litres of air approximately weigh 2.3 g. The lack in patient specificity is furthermore minimised by the comparable mean age (58 ± 13.4 years vs. 64 ± 13 years) of both study populations. Yet still the incongruences of both study populations and the estimative character of the formula by Cressoni et al. bear potential inaccuracies which automatically apply to ELW.

Another limitation of this study might be lung segmentation. Depending on the level of atelectasis automated lung segmentation was not always possible [23]. While in this study subsequent manual segmentation correction was performed only by a single person, it could generally bear the risk of an increased interrater variability. In order to standardise the acquisition of CT-based parameters and even more to ensure a fast determination of these parameters in clinical use, ARDS-specific lung analysis software would be desirable. In this regard, recent developments in the field of neuronal networks seem very promising [24].

## Conclusion

Excess lung weight (ELW) could serve as a non-invasive parameter to determine the amount of pulmonary oedema. Due to its strong association to a patient’s mean SOFA and SAPS II score, it could be regarded as an early radiological marker of ARDS severity. To clarify whether this radiological marker also bears any potential to predict patient outcome, further studies will be necessary.

## Supporting information

S1 File Ventilation and individual PEEP trial. (PDF)

S2 File Demographic characteristics, quantitative CT and clinical parameters of the study population. (PDF)

S1 Data Minimal data set. Abbreviations: SD *=* standard deviation; BSA *=* body surface area; FiO2 *=* fraction of inspiratory oxygen; PaO2 *=* arterial partial pressure of oxygen; IBW *=* ideal body weight; PEEP *=* positive end-expiratory pressure; Ppeak *=* peak (inspiratory) pressure; Pdrive *=* driving pressure; Pmean *=* mean pressure; Pplat *=* plateau pressure; SV *=* stroke volume; SVI *=* stroke volume index; SVRI *=* systemic vascular resistance index; CO *=* cardiac output; CI *=* cardiac index; ITBV *=* intrathoracic blood volume; ITBVI *=* intrathoracic blood volume index; EVLW *=* extravascular lung water; EVLWI *=* extravascular lung water index; BE *=* base excess; CaO2 *=* arterial concentration of oxygen; PAO2 *=* alveolar partial pressure of oxygen; AaDO2 *=* alveolar-arterial oxygen gradient. *Calculations and correlations were conducted in mmHg (1mmHg ≈ 133.32 Pa) and cmH2O (1cmH2O ≈ 98.07 Pa).
(XLSX)

## List of abbreviations

qCT: quantitative computed tomography
ARDS: acute respiratory distress syndrome
ICU: intensive care unit
ELW: excess lung weight
EVLW: extravascular lung water
PaO_2_/FiO_2_: arterial partial pressure of oxygen/ fraction of inspirational oxygen (oxygenation index
PEEP: positive end-expiratory pressure
SOFA: Sequential Organ Failure Assessment Score
SAPS II: Simplified Acute Physiology Score II
PiCCO: pulse contour cardiac output
ICC: intra-class correlation coefficient
CI: cardiac index
ITBVI: intrathoracic blood volume index
ECMO: extracorporeal membrane oxygenation
HU: Hounsfield unit
fRC: functional residual capacity
