# Supplementary material for: A quantitative CT parameter for the assessment of pulmonary oedema in patients with acute respiratory distress syndrome
Source: PLoS One. 2020 Nov 12;15(11):e0241590. doi: 10.1371/journal.pone.0241590 (PMC7660563; doi:10.1371/journal.pone.0241590)
Supplement: S1 File — (PDF) [file pone.0241590.s001.pdf]

S1 Doc. Ventilation and individual PEEP trial

All patients were ventilated with an Engström Carestation ventilator (GE Healthcare, Munich, Germany). Ventilation was conducted in the volume-control mode with a tidal volume (V_T) of 6 ml/kg ideal body weight. Respiratory rate (RR) was set to keep arterial pH above 7.2.

Per protocol of our intensive care unit (ICU) all patients underwent a recruitment manoeuver according to current recommendations [1] in order to standardise lung volume history [2]. Ventilator mode was switched to the pressure-controlled mode and PEEP was slowly increased from the individual baseline level to 35 cm H_2O over a time period of 5 minutes while the difference between end-inspiratory and end-expiratory tracheal pressure was kept at 15 cmH_2O. After a 2-minute recruitment period, the ventilator was returned to volume-controlled mode with a V_T of 6 ml/kg ideal body weight and a PEEP exceeding the initial setting by 6 cm H_2O. All the other ventilator settings were kept constant. Measurements were taken in 10-minute intervals to allow equilibration of gas exchange, haemodynamics, and lung mechanics, before PEEP was reduced stepwise by 2 cm H_2O. The decremental PEEP trial was stopped when Estat,RS did not further decrease by the reduction of PEEP ($PEEP_{Estat,RS}$) and ventilation was continued with this setting. Moreover, patients were paralysed with cis-atracurium [3] and sedated with midazolam (5 to 15mg/h) and fentanyl (0.5 to 2.5mg/h) in order to achieve a Richmond Agitation-Sedation Score (RASS) of -5 for the duration of the CT scans [4].

Subsequent to PEEP titration the CT scans were conducted as described in the main manuscript.

References:

1. Keenan SP, Sinuff T, Burns KEA, Muscedere J, Kutsogiannis J, Mehta S, et al. Clinical practice guidelines for the use of noninvasive positive-pressure ventilation and noninvasive continuous

positive airway pressure in the acute care setting. CMAJ : Canadian Medical Association Journal. 2011;183(3):E195-E214. doi: 10.1503/cmaj.100071. PubMed PMID: PMC3042478.

2. Nishida T, Suchodolski K, Schettino GP, Sedeek K, Takeuch M, Kacmarek RM. Peak volume history and peak pressure-volume curve pressures independently affect the shape of the pressure-volume curve of the respiratory system. Critical care medicine. 2004;32(6):1358-64. Epub 2004/06/10. PubMed PMID: 15187520.

3. Papazian L, Forel JM, Gacouin A, Penot-Ragon C, Perrin G, Loundou A, et al. Neuromuscular blockers in early acute respiratory distress syndrome. The New England journal of medicine. 2010;363(12):1107-16. Epub 2010/09/17. doi: 10.1056/NEJMoa1005372. PubMed PMID: 20843245.

4. Ely EW, Truman B, Shintani A, Thomason JW, Wheeler AP, Gordon S, et al. Monitoring sedation status over time in ICU patients: reliability and validity of the Richmond Agitation-Sedation Scale (RASS). Jama. 2003;289(22):2983-91. Epub 2003/06/12. doi: 10.1001/jama.289.22.2983. PubMed PMID: 12799407.
