# Supplementary material for: A quantitative CT parameter for the assessment of pulmonary oedema in patients with acute respiratory distress syndrome
Source: PLoS One. 2020 Nov 12;15(11):e0241590. doi: 10.1371/journal.pone.0241590 (PMC7660563; doi:10.1371/journal.pone.0241590)
Supplement: S2 File — (PDF) [file pone.0241590.s002.pdf]

S2 Table. Demographic characteristics, quantitative CT and clinical parameters of the study population

Parameter	total		males		females		P value
	mean	SD	mean	SD	mean	SD	
**Demographic characteristics**							
Patients	28		20		8		
Age [years]	58	13	59	15	55	9	0.38
Height [cm]	175	7	176	7	171	7	0.1
Weight [kg]	94	23	95	26	92	10	0.72
Body mass index [kg/m²]	31	7.9	30.7	9.1	31.6	3.1	0.7
Body surface area [m²]	2.1	0.2	2.1	0.2	2	0.1	0.21
**Ventilation**							
Total lung volume [ml]	3900.5	1324.1	4272.9	1206.1	2969.5	1134	0.02
Volume/BSA [ml/m²]	1877	682.2	2040.1	647.2	1469.3	590.7	0.052
Lung gas volume [ml]	1957.5	1032.8	2152.8	1045	1469.4	818.6	0.1
Lung gas volume [%]	0.5	15.6	0.5	16	0.49	14.6	0.91
Lung tissue volume [ml]	1942.9	738.7	2120.1	723.4	1500.1	571.8	0.04
Lung tissue volume [%]	0.5	15.62	0.5	16.01	0.51	14.56	0.91
Gas tissue ratio	1.01	0.62	1.02	0.66	0.98	0.50	0.72
Mean lung density [HU]	-482.9	156.2	-485.1	160.1	-477.3	145.6	0.91
Percentile 50	-540.1	261.7	-548.9	255.2	-518	276.1	0.8
lung compartments							
Over-inflated tissue [%]	3.8	7.7	5	8.8	0.7	1.1	0.048
Well inflated tissue [%]	53.5	20	51.8	20.1	57.7	18.9	0.5
Poorly inflated tissue [%]	16	7.3	15.99	7	16.04	8.2	0.99
Not inflated tissue [%]	26.7	17.4	27.2	16.4	25.5	19.5	0.85
Over-inflated tissue [ml]	206	468.4	277.2	537	28.1	50.7	0.06
Well inflated tissue [ml]	2103.6	1027.7	2240.5	1019.3	1761.3	967.1	0.29
Poorly inflated tissue [ml]	585.7	261.8	652.9	258.1	417.6	183.7	0.02
Not inflated tissue [ml]	1005.1	681.8	1102.3	678.7	762.2	626.7	0.25
Inflated tissue [%]	69.5	17.2	67.8	15.7	73.8	19.6	0.49
Inflated tissue [ml]	2689.3	1015.7	2893.4	962.2	2179.0	965.2	0.12
**Lung weight**							
Lung mass [g]	1945.8	738.8	2123.2	723.3	1502.2	572.2	0.04
Estimated lung weight [g]	1046.5	116.5	1070.9	110.2	985.5	109.4	0.1
Excess lung weight [g]	899.3	756	1052.4	758.1	516.8	599	0.08
Not inflated tissue [%]	26.7	17.4	27.2	16.4	25.5	19.5	0.85
Not inflated tissue [ml]	1005.1	681.8	1102.3	678.7	762.2	626.7	0.25
Under-inflated tissue [%]	42.7	20.5	43.2	21.1	41.6	19	0.86
Under-inflated tissue [ml]	1590.8	827.7	1755.2	851.1	1179.8	592.2	0.07

Parameter	total		males		females		P value
	mean	SD	mean	SD	mean	SD	
**Clinical parameters**							
Initial parameters							
$FiO_{2\ initial}$	52.4	15.9	54.4	17.1	47.5	10.9	0.24
$PaO_{2\ initial}$ [mmHg]*	77.4	8.5	77.8	9.4	76.4	5.6	0.66
$PaO_2/FiO_{2\ initial}$ [mmHg]*	160.2	44.8	155.8	42.4	171.2	48.5	0.47
Ventilator settings							
Tidal volume [ml]	408.1	77.8	424.8	72.5	360.7	73	0.09
Tidal volume/ kg IBW [ml]	5.9	1.11	5.98	1.18	5.68	0.82	0.5
Respiratory rate [1/min]	18.5	4.2	18.9	4.2	17.8	4.1	0.55
$FiO_{2\ CT}$	50.4	16.7	53.5	17.2	42.5	12.2	0.09
$PaO_2/FiO_{2\ CT}$ [mmHg]	188	72	178.4	72.5	212	64.8	0.28
Applied ventilation pressures							
PEEP [cmH_2O]*	13.2	4.1	13.4	4.1	12.7	4.0	0.69
Ppeak [cmH_2O]*	29.2	6.7	29.6	5.8	28.1	8.4	0.67
Pdrive [cmH_2O]*	13.4	4.9	13.5	4.9	13.1	4.8	0.86
Pmean [cmH_2O]*	17.7	4.0	17.9	3.6	17.1	4.7	0.68
Pplat [cmH_2O]*	26.6	5.4	26.9	4.6	25.8	7.1	0.7
Haemodynamics							
Heart rate [1/min]	98.9	15.9	100.2	16.3	95.5	14.4	0.49
Catecholamines [µg/kg × min]	0.15	0.29	0.14	0.29	0.17	0.29	0.85
Systolic pressure [mmHg]*	126	17.1	122.4	15.4	135.1	17.5	0.12
Diastolic pressure [mmHg]*	63	10.7	60.5	9.4	69.1	11.2	0.1
Mean arterial blood pressure [mmHg]*	82.3	11.7	79.5	7.8	89.4	15.9	0.15
PiCCO® measurements							
SV [ml]	74.2	17	71.6	15	79.6	19.4	0.35
SVI [ml/m^2]	36.2	9.8	33.5	7.4	41.9	11.5	0.11
SVRI [$dyn \times s/cm^5 \times m^2$]	1889.2	470.5	1845.9	444.2	1981.3	510	0.55
CO [l/min]	7.6	2.1	7.6	2.1	7.5	1.9	0.84
CI [$l/min/m^2$]	3.6	1	3.6	0.9	3.7	1.2	0.79
ITBV [ml]	1661.8	368.1	1746.3	382.8	1482.3	254	0.07
ITBVI [ml/m^2]	900.6	183.3	915.9	194.3	866	149.8	0.51
EVLW [ml]	1021.2	479.8	1123.8	534.8	790.4	166.2	0.03
EVLWI [ml/m^2]	14.6	6.2	15.3	7	13	2.8	0.25

Parameter	total		males		females		P value
	mean	SD	mean	SD	mean	SD	
Blood gas analysis (arterial)							
$PaO_{2\,CT}$ [mmHg]*	87.1	29.6	88.6	34.3	83.2	10.5	0.54
$PaCO_2$ [mmHg]*	57	14	58.7	13	52.6	15.5	0.37
pH	7.35	0.08	7.34	0.08	7.35	0.07	0.84
SaO_2 [%]	94.5	2.9	94.3	2.9	94.9	2.9	0.62
HCO_3 [mmol/l]	29.8	8.3	30.4	8.1	28.4	8.7	0.59
BE [mmol/l]	4.5	7.7	4.9	7.5	3.4	8.1	0.67
Haemoglobin [g/dl]	9.3	1.4	9.4	1.5	9.2	1.1	0.78
Lactate [mmol/l]	1.01	0.46	1.03	0.35	0.97	0.66	0.82
Calculated values							
CaO_2 [ml/dl]	12.3	2	12.3	2.1	12.2	1.5	0.85
PAO_2 [mmHg]*	292	114.4	312.4	118.7	241.2	83.4	0.11
$AaDO_2$ [mmHg]*	204.9	110.9	223.7	113.9	158	86.8	0.14
Scores							
GCS_{CT}	5.3	3.9	5.8	4.2	3.8	1.9	0.17
SAPS II $_{CT}$	43.3	12.8	47.4	12.1	33.5	8.3	<0.01
SAPS II $_{mean}$	39.7	10.7	41.7	10.8	34.5	8.5	0.09
SAPS II $_{max}$	51.9	12.6	54.4	11.4	45.8	13.4	0.16
TISS 10 $_{CT}$	24.8	5.3	24.4	5.6	25.8	4.4	0.54
TISS 10 $_{mean}$	19.5	4.2	18.2	3.9	22.7	3.4	0.01
TISS 10 $_{max}$	28.6	4.6	28.2	4.5	29.8	4.5	0.44
SOFA-Score							
SOFA $_{initial}$	10.5	3.7	11.2	4	8.9	1.9	0.06
SOFA_lung $_{initial}$	3.1	0.8	3.2	0.8	3	0.7	0.55
SOFA_cv $_{initial}$	3	1.6	3.1	1.6	2.8	1.6	0.68
SOFA $_{CT}$	11.7	5.1	12.4	5.2	10	4.6	0.29
SOFA_lung $_{CT}$	3.4	0.8	3.42	0.7	3.25	0.8	0.64
SOFA_cv $_{CT}$	2.6	1.7	2.8	1.6	2	1.8	0.33
SOFA $_{mean}$	9.9	4.7	10.2	5.1	9.2	3.5	0.56
SOFA_lung $_{mean}$	3	0.8	2.9	0.7	3.1	0.8	0.6
SOFA_cv $_{mean}$	2.2	1.4	2.3	1.4	1.9	1.6	0.63
SOFA $_{max}$	13	5	13.7	5.1	11.5	4.4	0.31

Abbreviations: SD = standard deviation; BSA = body surface area; FiO_2 = fraction of inspiratory oxygen; PaO_2 = arterial partial pressure of oxygen; IBW = ideal body weight; PEEP = positive end-expiratory pressure; Ppeak = peak (inspiratory) pressure; Pdrive = driving pressure; Pmean = mean pressure; Pplat = plateau pressure; SV = stroke volume; SVI = stroke volume index; SVRI = systemic vascular resistance index; CO = cardiac output; CI = cardiac index; ITBV = intrathoracic blood volume; ITBVI = intrathoracic blood volume index; EVLW = extravascular lung water; EVLWI = extravascular lung water index; BE = base excess; CaO_2 = arterial content of oxygen; PAO_2 = alveolar partial pressure of oxygen; $AaDO_2$ = alveolar-arterial oxygen gradient. *Calculations and correlations were conducted in mmHg (1mmHg ≈ 133.32 Pa) and cmH_2O ($1cmH_2O$ ≈ 98,07 Pa).

This table presents the mean values and standard deviations of all parameters used in this study (for male/ female patients and the entire study population respectively). Furthermore it displays the assignment of the different CT-based parameters to three groups: ventilation, lung weight and perfusion.
Physiological parameters served as a counterpart in correlation analysis and for this purpose were also divided into subject-related groups. If not indexed differently all these parameters were collected at the time of CT acquisition.
Finally all status and prognosis scores collected for every patient and on each day of their stay on our intensive care unit (ICU) are presented at the bottom of the table.

An explanation/derivation of all parameters can be found below:

Ventilation:

If lung segmentation is completed, a histogram of all selected voxels (Figure 1 in main manuscript) can be compiled and used for the determination of all ventilation based parameters. Due to a constant volume of every voxel in a CT image it is possible to convert any amount of voxels into the respective volume.

Consequently **total lung volume [ml]** can be defined as the area under the curve (AUC) in a given histogram. However, this value does not equal the exact amount of gas as the segmented volume of interest also contains solid structures (bronchioli, alveoles, etc.) and lung compartments with differing levels of lung aeration. Over the years several authors [1-4] established thresholds for the differentiation between those levels of aeration: **Over-inflated tissue** (<-900 HU), **well inflated tissue** (-900 - -500HU), **poorly inflated tissue** (-500 - -100) and **not inflated tissue** (>-100) can thus be presented as both absolute volumes [ml] and relative percentages of the total lung volume [%]. For our study we defined the amount of **inflated tissue** as the sum of well inflated and poorly inflated tissue.

Mean lung density (MLD), the arithmetic mean of the histogram and **percentile 50**, the lung density below which 50% of all voxels fall, constitute other ways to indirectly describe lung aeration. In contrast to the latter though, MLD enables the computation of further ventilation parameters:

Under the assumption that lungs are composed by only two materials, gas (-1000 HU) and lung tissue/water (0 HU), MLD indicates the proportions in which both materials occur [5]. For instance a MLD of -750 HU theoretically implies that the entire lung consists of 75% gas and 25% tissue/water. **Lung gas volume [ml]** and **lung tissue volume [ml]** can thus be computed as a fraction of total lung volume according to the following equations [5, 6]:

$$gas\ volume = \frac{MLD}{-1000} \times total\ volume$$

$$tissue\ volume = \frac{1000 + MLD}{1000} \times total\ volume$$

Thereby the term $\frac{MLD}{-1000}$ represents the relative **lung gas volume [%]** while $\frac{1000+MLD}{1000}$ equals the relative **lung tissue volume [%]**.

The **gas tissue ratio** provides another possibility to portray current lung status [7]:

$$gas\ tissue\ ratio = \frac{gas\ volume}{tissue\ volume}$$

Compliant with the thresholds for different levels of lung aeration, not inflated (<0.1), poorly inflated (0.1 to 1), well inflated (1 to 9) and over-inflated lung tissue (> 9) can be distinguished by means of only one variable. For instance a lung with a MLD of -750 HU would result in a gas tissue ratio of 3 and thus well inflated tissue would presumably be its dominating level of lung aeration.

Lung weight:

To our knowledge the relation between CT number [HU] and physical density (ρ) [g/cm^3] was first examined in 1984 [8]. Back then Mull et al. described an almost linear relation for lung densities between -700 HU to -100 HU which for our purpose - the assessment of lung weight - is almost ideal. Furthermore as in their study physical density of frozen meat (0 HU; 1,02g/cm^3) almost resembled water (0 HU; 1g/cm^3), the bias of equating lung tissue and water in our calculation is negligible.

With that knowledge and the assumption that lung consists of only water/lung tissue (0 HU) and gas (- 1000HU) it is then possible to directly convert lung tissue volume [ml] into **lung tissue weight** [g] [5, 9].

$$lung\ tissue\ weight\ [g] = \ lung\ tissue\ volume\ [ml] \times \ 1[g/ml]$$

Gattinoni et al [5] neglected the weight of air for their calculation of **lung mass [g]**. However, in our correlations lung mass [g] (i.e. the sum of the weight of air and lung tissue weight) provided better results than lung tissue weight [g] on its own which is why we decided to present it in the table above.

$$lung\ mass\ [g] = lung\ tissue\ weight\ [g] + lung\ gas\ volume\ [ml] \times \rho(air)_{\mathrm{BTPS}}$$

BTPS: body temperature, ambient pressure, saturated (with water vapour)

Estimated lung weight [g] refers to a formula by Cressoni et al [10]: They determined lung weight in 100 CT images and developed a formula for the estimation of lung weight based on height in healthy people. Interestingly the mean age of their study population (64 ±13 years) resembled the mean age in our study. Although CT acquisition in their study was performed at full inspiration (while our patients were scanned in end-expiration), we decided to regard the little difference in gas weight as negligible and used their formula to calculate the **excess** of **lung weight [g]** (ELW):

$$excess\ lung\ weight = lung\ mass\ [g] - estimated\ lung\ weight\ [g]$$

Not inflated tissue as a measurement of atelectasis and hence a possible parameter for the assessment of lung weight was also assigned to the group of lung weight parameters. Likewise we examined the amount of **under-inflated tissue** (i.e. the sum of not and poorly inflated tissue) as a parameter within the lung weight group.

Clinical parameters:

If not indexed differently all clinical parameters used in this study were collected at the time of CT acquisition. Only the fraction of inspiratory oxygen (**FiO_2**) and the arterial partial pressure of oxygen (**PaO_2**) were additionally determined on admission for the calculation of **initial PaO_2/FiO_2**.

Ventilator settings and applied ventilation pressures were gathered as displayed on the ventilator screen, for the determination of haemodynamics, pulse contour cardiac output technology (PiCCO®) measurements and blood gas parameters an arterial line was necessary.

The content of arterial oxygen (**CaO_2**), the alveolar partial pressure of oxygen (**PAO2**) and the difference between the two of them (**$AaDO_2$**) were computed as follows:

$$CaO2\ [ml/dl] = SaO2 \times Hb\ [g/dl] \times 1.39\, ml/g + (PaO2\ [mmHg] \times 0.0031\ [ml/mmHg \times dl])$$

$$PAO2\ [mmHg] = FiO2 \times (760mmHg - 47mmHg) - \frac{PaCO2\ [mmHg]}{RQ}$$

$$AaDO2 = PAO2 - PaO2$$

Note: We used a respiratory quotient (RQ) of 0.85.

Status and prognosis scores:

Several status and prognosis scores were determined for every patient on each day during their stay on intensive care unit (ICU):

Glasgow Coma Scale (GCS), originally introduced for patients after traumatic head injury, served as a parameter for the assessment of a patient's conscious state [14].
Therapeutic Intervention Scoring System 10 (TISS 10) is a complexity score to measure the level of expense and effort necessary for the patient's treatment on ICU [15, 16].
Simplified Acute Physiology Score (SAPS II) was introduced to grant a precise assessment of a patient's physiological state [17]. Together with TISS 10 it is currently used to calculate reimbursement for a patient's treatment on ICU in German hospitals.

Sequential Organ Failure Assessment -Score (SOFA) can be used to describe a patient's physiological state throughout the stay on ICU and is closely associated with a patient's outcome [18]. It consists of six subcategories (respiratory system, nervous system, cardiovascular system, liver, coagulation and kidneys). As respiratory system and cardiovascular system both play an important role in ARDS pathophysiology, these SOFA subcategories were additionally regarded as individual parameters and indexed as '**lung**' and '**cv**'.

Further indices used in connection with status and prognosis scores are explained here:

mean : average score over a patient's stay on ICU
max: maximum score over a patient's stay on ICU
initial: determined on admission on ICU
CT: determined at the time of CT acquisition

References

[1] L. Gattinoni, A. Pesenti, L. Avalli, F. Rossi, M. Bombino, Pressure-volume curve of total respiratory system in acute respiratory failure. Computed tomographic scan study, The American review of respiratory disease 136(3) (1987) 730-6.

[2] J.J. Rouby, L. Puybasset, A. Nieszkowska, Q. Lu, Acute respiratory distress syndrome: lessons from computed tomography of the whole lung, Critical care medicine 31(4 Suppl) (2003) S285-95.

[3] D. Chiumello, A. Marino, M. Brioni, I. Cigada, F. Menga, A. Colombo, F. Crimella, I. Algieri, M. Cressoni, E. Carlesso, L. Gattinoni, Lung Recruitment Assessed by Respiratory Mechanics and Computed Tomography in Patients with Acute Respiratory Distress Syndrome. What Is the Relationship?, American journal of respiratory and critical care medicine 193(11) (2016) 1254-63.

[4] P. Klapsing, P. Herrmann, M. Quintel, O. Moerer, Automatic quantitative computed tomography segmentation and analysis of aerated lung volumes in acute respiratory distress syndrome-A comparative diagnostic study, Journal of critical care 42 (2017) 184-191.

[5] L. Gattinoni, A. Pesenti, M. Bombino, S. Baglioni, M. Rivolta, F. Rossi, G. Rossi, R. Fumagalli, R. Marcolin, D. Mascheroni, et al., Relationships between lung computed tomographic density, gas exchange, and PEEP in acute respiratory failure, Anesthesiology 69(6) (1988) 824-32.

[6] P. Caironi, E. Carlesso, L. Gattinoni, Radiological imaging in acute lung injury and acute respiratory distress syndrome, Seminars in respiratory and critical care medicine 27(4) (2006) 404-15.

[7] M. Cressoni, P. Cadringher, C. Chiurazzi, M. Amini, E. Gallazzi, A. Marino, M. Brioni, E. Carlesso, D. Chiumello, M. Quintel, G. Bugedo, L. Gattinoni, Lung inhomogeneity in patients with acute respiratory distress syndrome, American journal of respiratory and critical care medicine 189(2) (2014) 149-58.

[8] R.T. Mull, Mass estimates by computed tomography: physical density from CT numbers, AJR. American journal of roentgenology 143(5) (1984) 1101-4.

[9] A. Protti, G.E. Iapichino, M. Milesi, V. Melis, P. Pugni, B. Comini, M. Cressoni, L. Gattinoni, Validation of computed tomography for measuring lung weight, Intensive care medicine experimental 2(1) (2014) 31.

[10] M. Cressoni, E. Gallazzi, C. Chiurazzi, A. Marino, M. Brioni, F. Menga, I. Cigada, M. Amini, A. Lemos, M. Lazzerini, E. Carlesso, P. Cadringher, D. Chiumello, L. Gattinoni, Limits of normality of quantitative thoracic CT analysis, Critical care (London, England) 17(3) (2013) R93.

[11] G.M. Lu, Y. Zhao, L.J. Zhang, U.J. Schoepf, Dual-energy CT of the lung, AJR. American journal of roentgenology 199(5 Suppl) (2012) S40-53.

[12] X. Liu, L. Yu, A.N. Primak, C.H. McCollough, Quantitative imaging of element composition and mass fraction using dual-energy CT: three-material decomposition, Medical physics 36(5) (2009) 1602-9.

[13] K.D. Hagspiel, L. Flors, A.M. Housseini, A. Phull, E. Ali Ahmad, U. Bozlar, P.T. Norton, H.J. Bonatti, Pulmonary blood volume imaging with dual-energy computed tomography: spectrum of findings, Clinical radiology 67(1) (2012) 69-77.

[14] G. Teasdale, B. Jennett, Assessment of coma and impaired consciousness. A practical scale, Lancet (London, England) 2(7872) (1974) 81-4.

[15] D.R. Miranda, A. de Rijk, W. Schaufeli, Simplified Therapeutic Intervention Scoring System: the TISS-28 items--results from a multicenter study, Critical care medicine 24(1) (1996) 64-73.

[16] R. Lefering, Biostatistical aspects of outcome evaluation using TISS-28, The European journal of surgery. Supplement. : = Acta chirurgica. Supplement (584) (1999) 56-61.

[17] J.R. Le Gall, S. Lemeshow, F. Saulnier, A new Simplified Acute Physiology Score (SAPS II) based on a European/North American multicenter study, Jama 270(24) (1993) 2957-63.

[18] J.L. Vincent, R. Moreno, J. Takala, S. Willatts, A. De Mendonca, H. Bruining, C.K. Reinhart, P.M. Suter, L.G. Thijs, The SOFA (Sepsis-related Organ Failure Assessment) score to describe organ dysfunction/failure. On behalf of the Working Group on Sepsis-Related Problems of the European Society of Intensive Care Medicine, Intensive care medicine 22(7) (1996) 707-10.
